# Neurobehavioral symptom changes following bilateral two-level stellate ganglion blocks in patients with traumatic brain injury: a retrospective analysis of 102 patients

**DOI:** 10.3389/fnins.2026.1924374

**Published:** 2026-09-22

**Authors:** Sean W. Mulvaney, Sanjay Mahadevan, Kyle J. Dineen, Roosevelt J. Desronvilles, Jessi Truett

**Affiliations:** 1 Department of Emergency Medicine, Uniformed Services University, Bethesda, MD, United States; 2 Orthobiologics Research Initiative Inc, North Bethesda, MD, United States

**Keywords:** concussion, neurobehavioral symptoms, stellate ganglion, stellate ganglion block (SGB), traumatic brain injury, two-level cervical sympathetic chain block

## Abstract

**Introduction:**

Persistent neurobehavioral symptoms following traumatic brain injury (TBI) can substantially affect patient functioning, and effective treatment options remain limited. This study evaluated changes in neurobehavioral symptoms following bilateral two-level cervical sympathetic chain block (2LCSB) in patients with persistent TBI-related symptoms. A secondary objective was to examine whether changes in neurobehavioral symptoms differed by sex.

**Methods:**

A retrospective case series was conducted among 102 patients with TBI-related neurobehavioral symptoms of at least 3 months' duration who underwent ultrasound-guided bilateral 2LCSB between September 2022 and October 2024. Neurobehavioral Symptom Inventory (NSI) scores were assessed at baseline, 1 week, and 1 month following the procedure. The cohort included 69 males and 33 females. Changes in NSI total scores and subscores across assessment periods were evaluated using repeated-measures analysis of variance with Greenhouse–Geisser correction when the assumption of sphericity was not met. *Post hoc* comparisons were adjusted for multiple comparisons.

**Results:**

Of the 102 patients that underwent the bilateral SGB procedure, 92 had improvement in their NSI scores (90.19%) with 10 patients not reporting improvement (9.80%). Mean NSI total score decreased from 38.88 (SD, 14.96) at baseline to 21.03 (SD, 14.33) at 1 week and 20.75 (SD, 13.95) at 1 month. NSI total scores differed significantly across assessment periods, *F*_(1.64,165.59)_ = 102.26, *p* < 0.001, partial η^2^ =0.503. Compared with baseline, mean NSI total score decreased by 17.85 points at 1 week and 18.14 points at 1 month (both adjusted *p* < 0.001). Scores did not differ significantly between 1 week and 1 month (adjusted *p* = 0.789), indicating maintenance of the observed reduction through 1 month. NSI subscores demonstrated a similar pattern, with a significant effect of time, *F*_(1.47,148.09)_ = 35.83, *p* < 0.00. Although males and females demonstrated descriptive reductions in NSI scores following 2LCSB, there was no significant evidence that the longitudinal pattern of change differed by sex. The average NSI scores across 102 patients showed notable improvements in patient reported symptoms with an average of 43% improvement in TBI-related neurobehavioral symptoms.

**Discussion:**

The use of bilateral Two-Level Cervical Sympathetic Chain Block (2LCSB) may be helpful in providing short term neurobehavioral symptoms for patients' relief from prior TBI's based on NSI scores. 2LCSB may improve chronic TBI symptoms through this minimally invasive interventional procedure, although further validated studies are needed to establish causality.

## 1 Introduction

Traumatic Brain Injuries (TBI) affect roughly 1.7–3.8 million people annually within the United States and over 50 million cases globally (Bell et al., 2017; Maas et al., 2022). Additionally, TBIs rank as one of the costliest public health issues in the United States with expenses exceeding over 400 million United States Dollars (USD) per year and ranking amongst the top three causes of disability and death. Military populations are particularly affected, as an estimated 28% of military members are known to have TBI (Reger et al., 2022). Prior military research suggests that TBI increases rates of posttraumatic stress disorder (PTSD), worsens psychological and behavioral health conditions, quality of life, increases likelihood of suicide (Brenner et al., 2023) and brain cancer development (Stewart et al., 2024). While TBI is seen in a variety of demographic groups, males are generally considered at a higher risk overall (Haarbauer-Krupa et al., 2021). Sex may also contribute to differences in symptom presentation and recovery following TBI. Prior studies have identified sex-related differences in post-TBI outcomes, including neurobehavioral and post concussive symptoms, with female patients reporting greater symptom burden on the Neurobehavioral Symptom Inventory (NSI) in some populations (Babakhanyan et al., 2024; Gray et al., 2020; Merritt et al., 2022). These findings suggest that sex may represent an important source of variability when evaluating neurobehavioral outcomes following TBI and support consideration of sex-specific outcomes when assessing potential interventions. For this study, the study population was stratified by sex (male, female) to explore outcome differences which may inform future any changes to 2LCSB administration to patients with TBI. This stratification was done given prior evidence suggesting sex-based differences in TBI risk and outcome trajectories. Additionally, there are potential neuroprotection effects associated with hormonal, neuroinflammatory responses, and alternative microglial activity associated with females (Herson et al., 2009). If untreated, persistent TBI symptoms may become chronic and contribute to long-term cognitive degeneration and related disease (Monsour et al., 2022).

Mechanistically, TBIs are associated with neuron injury, damage to the blood-brain-barrier, chronic neuroinflammation, and mitochondrial dysfunction (Gentleman et al., 2004; Xiong et al., 1997). Neurobehavioral symptoms may present in a variety of aspects including somatic (headaches, dizziness), cognitive (memory and concentration), and affective (emotion deregulation) rapidly or develop gradually and may persist for months or years without resolution (Andriessen et al., 2010). However, symptoms and degree of injury may differ substantially between patients, and treatment options should be individually tailored to each patient's injury and circumstances. Since patients are often unable to recall the circumstances of their TBIs, an accurate clinician diagnosis is critical.

A major challenge in treating persistent TBI symptoms is the lack of effective interventions as over 30 clinical trials have failed to identify a consistently successful treatment (Xiong et al., 2018). The stellate ganglion block (SGB) is an injection procedure targeting the cervical sympathetic chain. It has been used to treat many disorders including posttraumatic stress disorder (PTSD), complex regional pain syndrome, and other pain-related conditions (Duong et al., 2018), as the stellate ganglia are hypothesized to participate in the sympathetic nervous system signaling (Rusu et al., 2025). As a procedure, SGB is largely considered a safe and well-accepted therapeutic intervention (McLean, 2015).

The term SGB can be somewhat inaccurate as all patients have cervical sympathetic trunks, but roughly 20% of patients are found to lack a stellate ganglion. Additionally, a 2020 study showed that a two-level cervical chain block at C4 and C6 provided superior symptom relief compared to a single block at C6 (Mulvaney et al., 2020). For these reasons, the term two level cervical sympathetic chain block (2LCSB) is considered more accurate nomenclature but is not widely acknowledged in medical literature, so the term SGB is often retained.

Mulvaney et al. (2010, 2014) first reported right-sided SGB at the 6th vertebrae as a therapeutic interventional treatment option for military-related PTSD, later supported by Rae Olmsted et al.'s (2020) multi-center randomized control trial (RCT) showing efficacy when treatments are spaced 2 weeks apart. The incorporation of bilateral 2LCSB was later adopted and performed on consecutive days to minimize risk associated with inadvertent bilateral recurrent laryngeal nerve block and related respiratory distress. Recent studies have further demonstrated durable benefits, with PTSD improvement lasting 6 months and anxiety reduction lasting 3 months (Mulvaney et al., 2025c,a). Additional studies have shown promising results and durability for using 2LCSB to treat patients with TBI (Mulvaney et al., 2024a,b, 2025b). While preliminary findings are promising, further mechanistic research is needed to clarify how 2LCSB produces these symptom changes. While not the focus or aim of this study, it is possible that 2LCSB can improve neurobehavioral TBI symptoms by affecting cerebral perfusion patterns in the brain (Strogulski et al., 2023; Gupta et al., 2005). The primary aim of this study was to determine if performing ultrasound-guided, bilateral, two-level stellate ganglion blocks (SGB), performed on subsequent days, improves neurobehavioral symptoms in patients with prior history of symptomatic possible (Traumatic Brain Injuries) TBI, as assessed by the Neurobehavioral Symptom Inventory (NSI). A secondary objective was to evaluate sex-based differences in outcomes during the same time period.

## 2 Methods

### 2.1 Design

This retrospective case series evaluated patients who underwent bilateral two-level cervical sympathetic chain block (2LCSB) for persistent neurobehavioral symptoms associated with a history of traumatic brain injury (TBI). The study was approved by the Institutional Review Board of the Institute of Regenerative and Cellular Medicine (IRCM-2024-413). The retrospective study period extended from September 2022 through October 2024.

### 2.2 Inclusion criteria

The retrospective cohort included 102 patients who underwent bilateral 2LCSB as part of routine clinical care and had persistent TBI-related neurobehavioral symptoms. Eligibility required: (1) a clinician-documented history consistent with TBI and associated neurobehavioral symptoms; (2) persistence of symptoms for at least 3 months; (3) physician evaluation before treatment; (4) a baseline Neurobehavioral Symptom Inventory (NSI) score ≥ 12; and (5) complete NSI assessments at baseline, 1 week, and 1 month following treatment.

Clinical histories were reviewed by a physician to determine whether patients had a history consistent with TBI based on available medical documentation, patient-reported injury history, and/or clinically relevant features such as loss of consciousness or memory disturbance. Because these data were obtained retrospectively from routine clinical practice, documentation regarding the mechanism of injury, associated injuries and comorbidities, and whether a formal TBI diagnosis had been made at the time of the original injury was not uniformly available.

The NSI was selected as the primary patient-reported outcome measure for evaluating neurobehavioral symptom burden associated with TBI. The instrument consists of 22 items assessing common post concussive symptoms across somatic, cognitive, affective, and sensory domains, with higher scores indicating greater symptom severity.

### 2.3 Exclusion criteria

Patients were not excluded on the basis of age, sex, education, occupation, or medical history. Patients with a baseline NSI score ≤ 11 or without complete outcome data at all prespecified assessment points were excluded. The NSI score of 11 is considered clinical useful for this study because there is research to suggest that an NSI score cutoff of 11 is indicative of patient's post-concussive symptoms transitioning from mild to moderate (Streicher et al., 2026).

### 2.4 Sample size

The sample size was determined by the number of eligible patients during the period between September 2022 and October 2024. No *a priori* sample-size calculation was performed because of the retrospective nature of the study. The study size was determined by the number of patients treated with bilateral 2LCSB during the study period who met all eligibility criteria and had complete outcome data at the prespecified assessment points.

### 2.5 Two-level cervical sympathetic chain block

All included patients underwent bilateral 2LCSB between September 2022 and October 2024. The clinical and procedural details of the 2LCSB technique have been described previously (Mulvaney et al., 2015). Briefly, under ultrasound guidance, 6–8 mL of 0.5% ropivacaine was administered adjacent to the cervical sympathetic chain at the level of the right sixth cervical vertebra (C6), followed by 1.5–2 mL of 0.5% ropivacaine at the level of the right fourth cervical vertebra (C4). The same procedure was performed on the contralateral side the following day. Staging the bilateral procedures on separate days was intended to reduce the risk of simultaneous bilateral recurrent laryngeal nerve blockade and associated airway compromise (Mulvaney et al., 2021).

Procedural success was assessed approximately 5 min after injection by the presence of clinical findings consistent with Horner syndrome, as evaluated by trained clinical personnel and the treating physician. All procedures were performed by a single fellowship-trained physician (S.M.) with experience performing more than 4,000 stellate ganglion blocks and 2LCSBs.

### 2.6 Neurobehavioral symptoms inventory (NSI)

The NSI was administered at baseline, 1 week, and 1 month following bilateral 2LCSB. Assessments were completed through secure electronic communication, paper forms, or an automated cloud-based data collection system. Only patients with complete NSI data at all three assessment points were included in the analytic cohort; consequently, there were no missing outcome data within the final study sample.

The primary outcome was change in total NSI score from baseline to post-treatment follow-up. In addition, an exploratory NSI subscore was calculated using items considered less overlapping with symptoms commonly assessed by the Posttraumatic Stress Disorder Checklist for DSM-5 (PCL-5), consistent with previous publications examining TBI-related symptom profiles (Mulvaney et al., 2024a, 2025b). The subscore included NSI items assessing dizziness, balance or coordination difficulties, headaches, nausea, visual problems, hearing difficulty, sensitivity to light, sensitivity to noise, altered taste or smell, and numbness or tingling. The maximum possible subscore was 40 points.

This exploratory subscore was examined to characterize changes in predominantly somatic, vestibular, and sensory symptoms following treatment while reducing the contribution of symptoms that may overlap with posttraumatic stress and other behavioral health conditions. This distinction was considered relevant because total NSI scores may be influenced by comorbid posttraumatic stress and psychological symptoms (Kreitzer et al., 2019).

This retrospective case-series was approved by the institutional review board of the Institute of Regenerative and Cellular Medicine (IRCM-2024-413). The retrospective review included 102 patients who received bilateral 2LCSB and had TBI-related neurobehavioral symptoms as determined by a baseline NSI score of 12 or higher. All patients included had their medical histories reviewed by a clinician and were determined to have suffered and/or currently possessed a TBI based on evidence of pertinent TBI symptoms such as loss of consciousness, memory loss, etc. The sample size was determined by the number of eligible patients during the period between September 2022 and October 2024.

Patients with a self-reported NSI score of 11 or lower were excluded from the study. Because the data reported here is from a clinical practice, apart from the previous criterion, there were no exclusion criteria. Patients were included based on the following criteria: a health provider's formal assessment of a history of TBI-related neurobehavioral symptoms; TBI-related symptom duration of at least 3 months; physician evaluation; and NSI score. The NSI is considered a viable questionnaire for TBI symptoms by the U.S. Department of Defense and the Department of Veterans Affairs and was the primary outcome measure chosen for this study. In each case, symptoms had been present for at least 3 months. Not all patients disclosed how their injury was sustained, related injuries and existing conditions, or if they received a formal diagnosis of TBI at the time of injury. There was no randomization into placebo and interventional groups since all patients were seen within the scope of regular clinical practice, as is the format for retrospective reviews. This study was designed as a retrospective case series of patients who underwent bilateral 2LCSB for persistent TBI-related neurobehavioral symptoms, the available clinical dataset did not contain a contemporaneous untreated or usual-care cohort assessed using the same NSI schedule. Patients who did not undergo 2LCSB were not systematically administered the NSI at baseline, 1 week, and 1 month as part of routine clinical care. Consequently, an untreated comparison group with comparable eligibility criteria, symptom duration, outcome measurement, and follow-up intervals could not be reliably constructed retrospectively. Patients were included in the retrospective cohort regardless of demographic factors (such as age, sex, education, profession, or past medical history).

Patients included in this case series underwent bilateral 2LCSB between September 2022 and October 2024. All patients completed the NSI patient-reported outcome measures at baseline (BL), 1 week (1W), and 1-month (1M) post-2LCSB. The NSI was provided to patients either through a secure email system, via paper copy, or through an automated cloud-based data collection system. To be included in the study, all data points were captured; therefore, there is no missing data from participants.

The clinical and procedural details of the 2LCSB technique used in this study have been previously published (Mulvaney et al., 2015). In sum, ultrasound guidance was used to inject the cervical sympathetic chain with 6–8 mL of 0.5% ropivacaine at the level of the right 6th cervical vertebra and 1.5–2 mL of 0.5% ropivacaine at the level of the right 4th cervical vertebra. This exact procedural technique was repeated on the left side on the following day for every patient in this study, with the intent of eliminating the risk of inadvertent bilateral blockade of the recurrent laryngeal nerve, which could potentially lead to airway compromise (Mulvaney et al., 2021). The success of each block was determined via confirmation of Horner's syndrome in each patient 5 min following each injection by certified clinic personnel and injecting physician. All procedures were performed by a single fellowship-trained physician (S.M.) who had performed over 4,000 SGBs and 2LCSBs.

As done in prior publications on TBI, NSI questions that do not overlap with traditional symptoms of PTSD as measured on the PCL-5 outcome measure were isolated (Mulvaney et al., 2024a, 2025b). A sub-score of the NSI's non-overlapping questions was generated and denoted as NSI sub-score, with the maximum possible score being 40 points. The non-overlapping questions identified as specific to TBI included: prior symptomatic history of dizziness, loss of balance and coordination, headaches, nausea, vision problems, hearing difficulty, sensitivity to light and noise, loss of smell and taste, and numbness or tingling of the body. This sub-score of the NSI was used to determine if the improvements measured may be due to improvements in TBI-related neurobehavioral symptoms vs. improvements in other co-morbid conditions, since higher scores of the NSI are correlated with PTSD and other behavioral disorders (Kreitzer et al., 2019).

## 3 Results

### 3.1 Statistical tests

For this study, descriptive statistics were used to summarize participant characteristics and outcome measures. Continuous variables were calculated as means with standard deviations. The average age of all participants was determined and reported. Additionally, the average male, female, and total NSI scores and NSI sub-scores were generated as descriptive statistics for this dataset. This evaluation provided a quantified measure of symptom burden and distribution within the study cohort.

### 3.2 Results and data

A total of 102 patients met the eligibility criteria and were included in the retrospective analysis. Participants had a mean age of 44.0 years (SD = 10.5; range, 19–71 years); 69 (67.6%) were male and 33 (32.4%) were female. Table 1 presents the participant demographic data. Complete Neurobehavioral Symptom Inventory (NSI) data were available for all 102 patients at baseline, 1 week, and 1 month.

**Table 1:** Participant characteristics of the study cohort (*N* = 102).

Characteristic	Overall (*N* = 102)
Age, years, mean (SD)	44.0 (10.5)
Age, years, median (IQR)	44.0 (36.0–51.0)
Age, years, range	19–71
Sex, *n* (%)	
Male	69 (67.6)
Female	33 (32.4)

Values are presented as mean (SD), median (interquartile range [IQR]), range, or *n* (%), as appropriate.

### 3.3 NSI total score

Mean NSI total score decreased from 38.88 (SD = 14.96) at baseline to 21.03 (SD = 14.33) at 1 week and 20.75 (SD = 13.95) at 1 month. Overall, 92 of 102 patients (90.2%) demonstrated improvement in NSI scores, whereas 10 patients (9.8%) did not demonstrate improvement.

The results of this data analysis can be seen in Figures 1–3 and Tables 1, 2.

**Figure 1:**
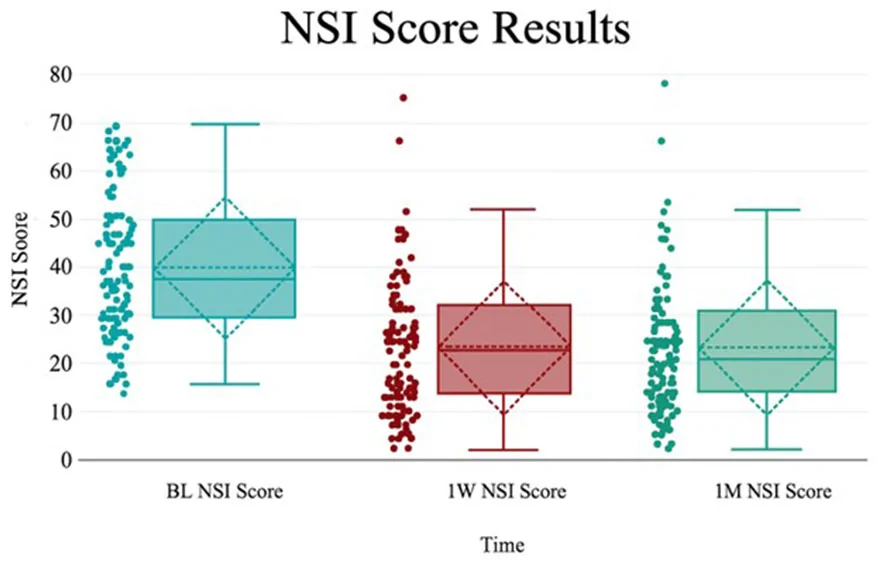
Box and whisker plot, depicting NSI scores for male, female, and all patients at baseline (BL), 1 week (1W), and 1 month (1M) after bilateral, two-level cervical sympathetic blockade (2LCSB). The middle line of each box at each time point represents the median NSI score. The top of each box represents the 75th percentile (Q3) NSI score. The bottom of each box represents the 25th percentile (Q1) NSI score. The height of each box represents the Interquartile Range (IQR), or Q3–Q1. The top whisker is the highest value NSI score within 1.5 times the IQR. The bottom whisker is the lowest value NSI score within 1.5 times the IQR. This box plot is overlayed on a dot plot of the raw individual data points to visualize the density of the NSI scores.

**Figure 2:**
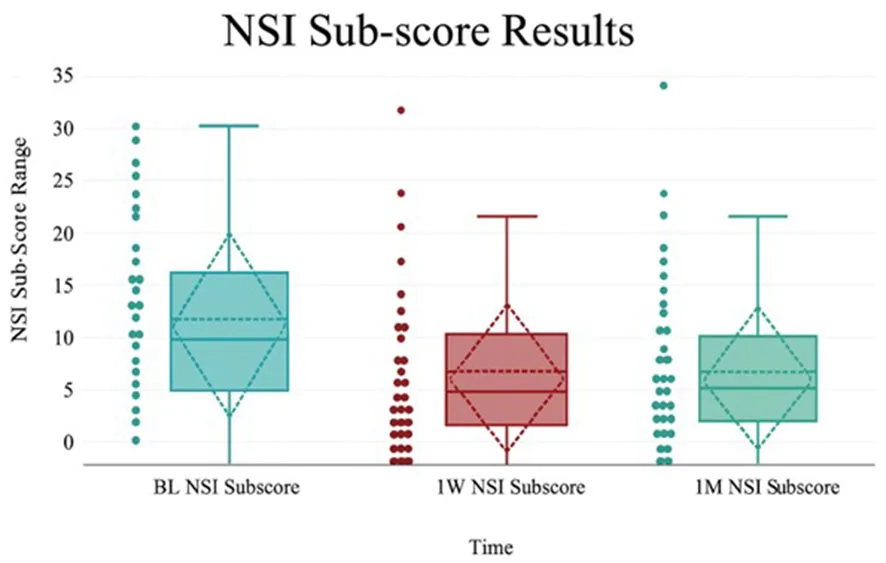
Box and whisker plot, depicting NSI sub-scores for male, female, and all patients at baseline (BL), 1 week (1W), and 1 month (1M) after bilateral, two-level cervical sympathetic blockade (2LCSB). The middle line of each box at each time point represents the median NSI sub-score. The top of each box represents the 75th percentile (Q3) NSI sub-score. The bottom of each box represents the 25th percentile (Q1) NSI sub-score. The height of each box represents the Interquartile Range (IQR), or Q3–Q1. The top whisker is the highest value NSI score within 1.5 times the IQR. The bottom whisker is the lowest value NSI sub-score within 1.5 times the IQR. This box plot is overlayed on a dot plot of raw individual data points to visualize the density of the NSI sub-scores.

**Figure 3:**
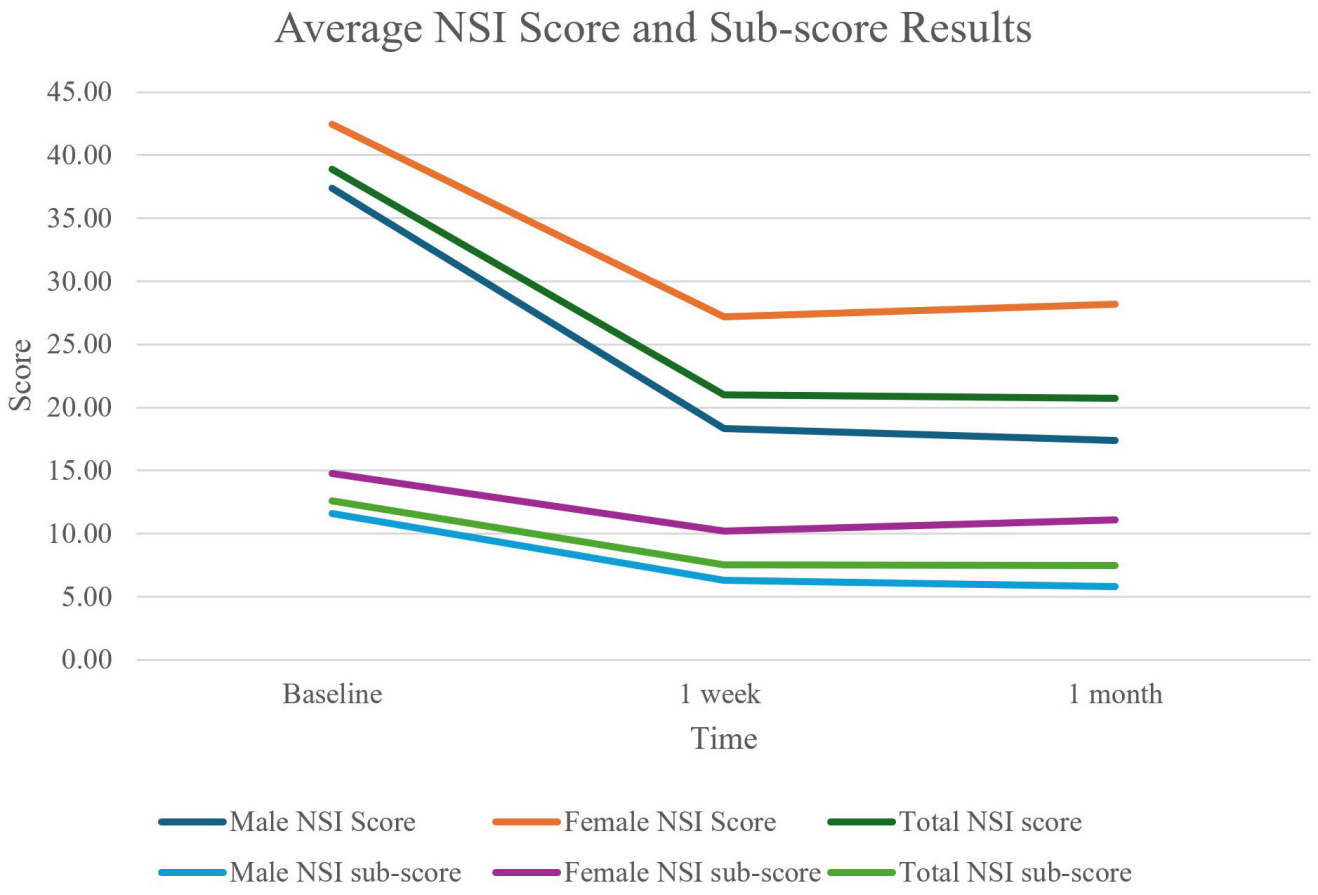
Line graph depicting average Male, Female, and Total NSI Score and average Male, Female, and Total NSI sub-score at Baseline (BL), 1-week (1W), and 1-month (1M) after 2LCSB.

**Table 2:** NSI scores, NSI sub-score scores, and percentage changes from baseline (BL), 1-Week (1W), and 1-Month (1M) following 2LCSB.

Sex subgroup	*n*	Baseline NSI, mean (SD)	1-Week NSI, mean (SD)	1 Month NSI, mean (SD)	Baseline sub-score, mean (SD)	1-Week sub-score, mean (SD)	1-Month sub-score, mean (SD)	% Change in NSI score
Female	33	42.45	27.18	28.21	14.79	10.21	11.09	33
Male	69	37.38	18.35	17.41	11.62	6.32	5.82	47
Total	102	38.88 (14.96)	21.03 (14.33)	20.75 (13.95)	12.70 (8.3)	7.37 (6.82)	7.23 (6.08)	43

## 4 Discussion

This study is the first large case series evaluating bilateral 2LCSB for treatment of TBI-related symptoms, as defined by NSI scores, and builds on three previously published case series (Mulvaney et al., 2024a,b, 2025b). The key aim of this paper is the evaluation of how 2LCSB improved NSI scores and the secondary aims included assessing sex-based outcomes.

Prior studies evaluated TBI-related neurobehavioral symptoms with and without related PTSD in patients. In this study, the NSI sub-score was used to assess changes in post-concussive neurobehavioral symptoms traditionally associated with TBI. Results showed a 41% overall sub-score improvement, with male patients improving by 50% compared to 25% in female patients. However, these findings should be interpreted with caution, as they may reflect symptom relief rather than underlying neurobiological changes.

Sex-based differences were observed in outcomes, with more males (69) than females (33) included in the dataset. Female patients had higher average baseline score, which may explain their lower percentage of improvement, but both groups improved at all intervals. While males generally are at a higher risk for TBI, some studies have suggested that female may experience superior outcomes due to hormonal neuroprotection, reduced neuroinflammatory responses, and alternative microglial activity (Herson et al., 2009). Future studies should consider block randomization to properly investigate sex-specific outcomes. Both the mean NSI scores and mean NSI subscores seem to plateau 1 week after treatment. While we are not certain as to the mechanistic cause of this, it appears that immediate symptom relief is achieved for most patients and that relief appears durable out to 1 month. Future studies are needed to confirm why further symptom-improvement does not appear to take place.

This study has limitations commonly associated with retrospective cohort reviews. While improvements are durable to 1 week and 1-month, the follow-up period is short and may not predict longer-term durability of improvements. Despite being a standard validated outcome measure, the NSI does not diagnose patients with TBI and higher NSI scores may correlate with comorbid pathologies such as PTSD (Kreitzer et al., 2019). Therefore, some of our findings rely partially on unvalidated analysis. Another limitation is our reliance on self-reported TBI symptoms in our findings. Self-reporting in TBI is limited by cognitive deficits associated with the injury, the stigma associated with both cognitive deficits and behavioral issues, and unclear terminology related to TBI (concussion, knocked out, etc.; Edmed et al., 2015). We recognize a key limitation is the use of an unvalidated NSI sub-score in an attempt to break out potential changes in neurobehavioral symptoms not usually associated with PTSD or anxiety. All patients in this study were characterized as having “possible” TBI due to history of chronic post-concussive symptoms. This can be considered a limitation because patients are often found to underreport their symptoms (Edmed et al., 2015; Port et al., 2002; Daugherty et al., 2023), which may have contributed to discrepancies in this data. Future studies should provide a more structured assessment of prior TBI history to accurately capture the target patient population in question.

Symptom improvements may be placebo effect mediated, which has been reported to be higher with injection procedures (de Craen et al., 2000). However, these findings were in reference to subcutaneous delivery of placebo migraine medication vs. oral delivery and may not be generalizable to sympathetic blocks which result in observable Horner's syndrome. Other minimally invasive musculoskeletal procedures can produce significant placebo effects (Holtedahl et al., 2015). However, other research suggests that one issue that continues to confound the results of many studies is comparing the tested substance to saline-injected control. There is evidence suggesting that a saline injection is not a control but may act as a treatment (Bar-Or et al., 2017).

Some research suggests dysautonomia vs. autonomic nervous system (ANS) dysfunction sequela to whiplash or cervical injury may contribute to neurobehavioral-related symptoms including sensory changes, musculoskeletal pain, headache, and sympathetic nervous system changes (Mourad et al., 2023; Sterling and Kenardy, 2006). It is possible these factors may be symptom generators not secondary to TBI symptoms, which we suspect the 2LCSB is modulating. We did not assess these factors within this study, but future studies may address if sympathetic chain blockade influences these variables. Finally, the use of a single keyer for data entry may call this data into question.

There is a notable gap in scientific understanding regarding the 2LCSB's mechanism of action and how it may alleviate TBI symptoms. Hypoxia, altered hemodynamics, and cerebral perfusion dysfunction are common after TBI and give rise to neuroinflammatory biomarkers markers following injury (Yan et al., 2014). Some research suggests damaged brain tissue, blood-brain-barrier (BBB) disruption, and mitochondrial dysfunction contribute to neuroinflammation via astrocytosis (Strogulski et al., 2023).

Severity and duration of the injury may cause microglial cells to alter their phenotypic functional activation state in response to TBI. Some studies have shown that neurobehavioral symptoms are associated with presence of pro-inflammatory cytokines in blood or cerebral spinal fluid (Malik et al., 2023). Finally, research has documented the presence of neurobehavioral symptoms after TBI up to or greater than 3 months after injury (Madhok et al., 2020). While the findings of this study do not directly address the presence of biofluid biomarkers or cerebral perfusion patterns, they do support an improvement in neurobehavioral symptoms up to 1 month after a TBI following 2LCSB. In our view, chronic hypoxia-induced glial cell activity, poor hemodynamics, and cerebral perfusion dysfunction directly contribute to TBI as a disease and may contribute to persistent neurobehavioral symptoms following injury.

Prior studies have suggested that SGB's mechanism may alter biomarkers traditionally associated with neuroinflammation and cerebral blood flow dysfunction, resulting in symptom improvement. This study did not include requisite methodologies to properly assess this theory, but other research has shown SGB to improve cerebral blood flow and perfusion patterns post-block (Gupta et al., 2005). In their 2025 study, Kostadinov and colleagues found SGB, when administered ipsilaterally to the most affected hemi-sphere at the C6 level using 8 mL of 0.5 levobupivacaine, to significant drop inflammatory biomarkers (IL-6, NSE, GFAP, among others) and intracranial pressure, along with improving oxygen supply to brain tissue in moderate and severe TBI patients (Kostadinov et al., 2025). In chronic TBI, there are persistent areas of decreasing cerebral perfusion (Vespa, 2016). These initial findings may support the hypothesis that neuroinflammatory markers associated with TBI are being altered after a cervical sympathetic block. The extent that hypoxia-induced microglial cell activation and astrogliosis is being either reversed or mediated, measured by improved hemodynamics and decreased inflammatory biomarkers, is unknown. However, future studies should include biomarker analysis and utilize rigorous methodological design to properly assess this. The results of this study are considered exploratory and descriptive rather than causal.

Lastly, a more robust exploration of patient history regarding their symptoms, including prior TBI diagnosis, length of time since injury, employment status, related pathologies (i.e., dysautonomia, cervical musculoskeletal injury, PTSD diagnosis), and circumstances of injury should be assessed, which was not possible in this cohort review.

## 5 Conclusion

This retrospective case series of 102 patients found bilateral ultrasound-guided 2LCSB to provide relatively durable improvements in neurobehavioral symptoms determined by decreases in NSI outcome scores after 1-week and 1-month. Ninety percent (90%) of patients reported improvements in symptoms. These findings only provide initial indications that bilateral 2LCSB may provide short-term relief for TBI-related neurobehavioral symptoms at 1-week and 1-month in patients with TBI's. Since TBI-related neurobehavioral pathologies are a significant cause of morbidity, especially in the military population, this procedure should be considered for further study. Future studies are needed to provide further understanding of the 2LCSB mechanism and its role in improving neurobehavioral-related injury symptoms.

## Data Availability

The data that support the findings of this study are available on request from the corresponding author. All data is freely accessible.
